# Should the CIWA-Ar be the standard monitoring strategy for alcohol withdrawal syndrome in the intensive care unit?

**DOI:** 10.1186/s13722-021-00226-w

**Published:** 2021-03-24

**Authors:** Tessa L. Steel, Shewit P. Giovanni, Sarah C. Katsandres, Shawn M. Cohen, Kevin B. Stephenson, Ben Murray, Hillary Sobeck, Catherine L. Hough, Katharine A. Bradley, Emily C. Williams

**Affiliations:** 1 Seattle-Denver Center of Innovation (COIN), VA Puget Sound Health Care System, Seattle Division, 1660 South Columbian Way S-152, SeattleSeattle, WA 98108 USA; 2 Division of Pulmonary, Critical Care, & Sleep Medicine, University of Washington, Seattle, WA USA; 3 Division of Pulmonary, Critical Care, & Sleep Medicine, Harborview Medical Center, Seattle, WA USA; 4 Department of Medicine, Harborview Medical Center, Seattle, WA USA; 5 University of Washington Internal Medicine Residency Program, Seattle, WA USA; 6 Department of Pharmacy Services, Harborview Medical Center, Seattle, WA USA; 7 Kaiser Permanente Washington Health Research Institute, Seattle, WA USA

**Keywords:** Alcohol-induced disorders, Nervous system, Critical care, Hypnotics and sedatives, Monitoring, physiologic, Drug monitoring, Quality of health care

## Abstract

**Background:**

The Clinical Institute Withdrawal Assessment for Alcohol-Revised (CIWA-Ar) is commonly used in hospitals to titrate medications for alcohol withdrawal syndrome (AWS), but may be difficult to apply to intensive care unit (ICU) patients who are too sick or otherwise unable to communicate.

**Objectives:**

To evaluate the frequency of CIWA-Ar monitoring among ICU patients with AWS and variation in CIWA-Ar monitoring across patient demographic and clinical characteristics.

**Methods:**

The study included all adults admitted to an ICU in 2017 after treatment for AWS in the Emergency Department of an academic hospital that standardly uses the CIWA-Ar to assess AWS severity and response to treatment. Demographic and clinical data, including Richmond Agitation-Sedation Scale (RASS) assessments (an alternative measure of agitation/sedation), were obtained via chart review. Associations between patient characteristics and CIWA-Ar monitoring were tested using logistic regression.

**Results:**

After treatment for AWS, only 56% (n = 54/97) of ICU patients were evaluated using the CIWA-Ar; 94% of patients had a documented RASS assessment (n = 91/97). Patients were significantly less likely to receive CIWA-Ar monitoring if they were intubated or identified as Black.

**Conclusions:**

CIWA-Ar monitoring was used inconsistently in ICU patients with AWS and completed less often in those who were intubated or identified as Black. These hypothesis-generating findings raise questions about the utility of the CIWA-Ar in ICU settings. Future studies should assess alternative measures for titrating AWS medications in the ICU that do not require verbal responses from patients and further explore the association of race with AWS monitoring.

## Text

Alcohol withdrawal syndrome (AWS) is common in patients admitted to intensive care units (ICUs) and can be fatal without individualized treatment [1]. Clinical guidelines therefore recommend use of a standardized, scaled measure to guide management of AWS [2]. The most widely used measure is the Clinical Instrument Withdrawal Assessment for Alcohol–Revised (CIWA-Ar), a 10-item scale based on patient interview and physical exam that showed clinical benefit in veterans admitted to a specialized detoxification unit with uncomplicated AWS [3, 4]. Studies in general hospital settings, however, show that 14–23% of patients in whom the CIWA-Ar is ordered cannot communicate and are not appropriate candidates for symptom-triggered therapy [5, 6]. The feasibility of using such a scale in ICUs, where patients are critically ill, often sedated, and frequently unable to speak due to encephalopathy or intubation (placement of a “breathing tube” in a patient’s upper airway to support breathing with a ventilator), has not been assessed. This study evaluated use of the CIWA-Ar in ICU patients with AWS at a hospital with a protocol specifying its use for assessment of AWS severity and response to treatment, and describes variation in CIWA-Ar monitoring across patient subgroups.

## Methods

This retrospective cohort study was designed to evaluate AWS treatment monitoring in medical, trauma, and neurological ICU patients at a large academic hospital. Data were collected from electronic hospital sources via automated extraction and subsequent manual chart review. The study was approved by the University of Washington Institutional Review Board.

All patients treated for AWS in the Emergency Department (ED) prior to ICU admission in 2017 were included. The sample was limited to ICU patients treated for AWS in the ED (as opposed to all ICU patients with AWS) to standardize the timing of AWS treatment onset. AWS was defined by primary or secondary discharge international classification of diseases (ICD) diagnosis codes and confirmed via manual chart review. Treatment for AWS was defined by continuous infusion or multiple administrations of benzodiazepines, antiepileptics, antipsychotics, antisympathomimetics, propofol, and/or ethanol intended to treat AWS, determined by review of clinical notes and pharmacy data. At the study hospital, ED providers initiate treatment for AWS on a case-by-case basis, using clinical experience to recognize signs/symptoms of AWS (there is no standardized screening protocol). Communication of clinical concern for AWS occurs via verbal physician-to-physician pass-off between ED and ICU providers. ICU providers can also recognize ED treatment for AWS by reviewing documentation of events that transpire in the ED using the electronic health record.

CIWA-Ar scores (0–67 points) were categorized as ≤ 8 mild, 9–19 moderate, and ≥ 20 severe [3]. Sedation monitoring within 24 h of ICU admission using the Richmond Agitation-Sedation Scale (RASS) was also evaluated. The RASS is a 10-point scale (-5 unarousable to + 4 combative) assigned by nurses or other providers that is widely used in ICU settings [7]. In patients with a documented RASS assessment, agitation/sedation was categorized as optimal (RASS − 2 to 0), oversedated (RASS -3 to -5), or undersedated (RASS + 1 to + 4) based on the Prevention and Management of Pain, Agitation/Sedation, Delirium, Immobility, and Sleep Disruption in Adult Patients in the ICU (PADIS) guidelines [8]. The PADIS guidelines are endorsed by all major international critical care societies and provide evidence-based recommendations regarding agitation/sedation goals. In ICUs at the study hospital, protocols for the CIWA-Ar (used specifically for AWS) and the RASS (used more generally for assessment of agitation/sedation) are triggered by a physician order. Nurses may decline to carry out the order for CIWA-Ar monitoring in patients who cannot respond to CIWA-Ar questions. While ICU nurses do not generally measure CIWA-Ar scores without a physician order, RASS assessments are sometimes performed at the discretion of ICU nurses without a physician order to document level of agitation/sedation. Admission severity of illness was determined by a proprietary algorithm used by the study hospital [i.e., 3 M All Patients Refined Diagnosis Related Groups (APR-DRG) grouper], including age, sex, admission diagnosis, and secondary conditions.

The main outcome was presence or absence of CIWA-Ar assessment(s) within 24 h of ICU admission [3, 7]. Analyses evaluated the proportion of patients with CIWA-Ar assessment(s) overall and across demographic and clinical characteristics. Variables with a significant univariate association with CIWA-Ar assessment (α set to 0.05) were further evaluated in a multivariable logistic regression model with adjustment for age, sex, race, intubation status (yes/no), and severity of illness, planned a priori*.* This was the first naturalistic evaluation of factors associated with CIWA-Ar monitoring in ICU patients. Given limited prior literature, predicting which variables would be most important to include in the multivariable model was challenging. We prioritized demographic characteristics and markers of illness severity (i.e., intubation status and the severity of illness APR-DRG grouper) a priori, then used univariate associations to guide inclusion of two additional patient characteristics: prior-year hospitalization with AWS and positive serum/urine alcohol level. Secondary analyses described AWS severity, as measured in patients with a documented CIWA-Ar assessment, and level of agitation/sedation, as measured in patients with a documented RASS assessment.

## Results

Among ICU patients treated for AWS (n = 97), only 56% (n = 54) were assessed with the CIWA-Ar within 24 h of admission (Table 1). Use of CIWA-Ar monitoring varied across several patient demographic and clinical characteristics (Table 2). In unadjusted analyses, CIWA-Ar assessments were less common among patients who were intubated (8%, n = 3/37), extremely ill (29%, n = 10/34), or Black (36%, n = 4/11), and more common among American Indian/Alaska Natives (91%, n = 10/11), patients previously hospitalized with AWS (84%, n = 16/19), and patients with a positive serum/urine alcohol level (65.5%, n = 38/58). After adjustment, only intubation status and race were significantly associated with CIWA-Ar assessment. Specifically, both intubation and Black race were associated with lower likelihood of CIWA-Ar monitoring than no intubation and White race, respectively (Table 2).

**Table 1:** Characteristics of intensive care unit (ICU) patients treated for alcohol withdrawal syndrome (AWS)

	All (n = 97)
Age years, mean (sd)	49.9 (12.0)
Male, no. (%)	79 (81.4)
Race, no. (%)	
American Indian/Alaska Native	11 (11.3)
Black	11 (11.3)
White	70 (72.2)
Other	5 (5.2)
Latino/Hispanic Ethnicity, no. (%)	11 (11.3)
Non-English primary language, no. (%)	7 (7.2)
Single, no. (%)	75 (77.3)
Homeless, no. (%)	26 (26.8)
Admission Severity of Illness, no. (%)	
Extreme	34 (35.1)
Major	37 (38.1)
Moderate	24 (24.7)
Minor	2 (2.1)
Intubated (“breathing tube” for mechanical ventilation)	37 (38.1)
Prior-year hospitalization with AWS, no. (%)	19 (19.6)
Positive serum/urine alcohol level,^a^ no. (%)	58 (59.8)
AWS was the primary reason for admission, no. (%)	5 (5.2)
Admitting service, no. (%)	
Medical ICU	49 (50.5)
Trauma ICU	24 (24.7)
Neurological ICU	24 (24.7)
Admission^b^ CIWA-Ar assessment, no. (%)	54 (55.7)
Admission^b^ CIWA-Ar category, no. (%)^c^	
Mild AWS (CIWA-Ar score ≤ 8)	28 (51.9)
Moderate AWS (CIWA-Ar score 9–19)	20 (37.0)
Severe AWS (CIWA-Ar score ≥ 20)	6 (11.1)
Admission^b^ RASS assessment, no. (%)	91 (93.8)
Admission^b^ RASS category, no. (%)^d^	
Oversedation (RASS score − 3 to − 5)	34 (37.4)
Optimal sedation (RASS score − 2 to 0)	31 (34.1)
Undersedation (RASS score + 1 to + 4)	26 (28.6)

*CIWA-Ar* Clinical Institute Withdrawal Assessment for Alcohol-Revised, *RASS* Richmond Agitation-Sedation Scale

^a^Assessment completed within 24 h of hospital presentation

^b^Assessment completed within 24 h of ICU admission

^c^Denominator used to calculate percentages was the number of patients with a CIWA-Ar assessment (n = 54)

^d^Denominator used to calculate percentages was the number of patients with a RASS assessment (n = 91)

**Table 2:** Unadjusted and adjusted prevalence of Clinical Institute Withdrawal Assessment for Alcohol-Revised (CIWA-Ar) monitoring across subgroups of intensive care unit (ICU) patients treated for alcohol withdrawal syndrome (AWS)

	CIWA-Ar Assessment^a^						
	No (n = 43) No. (Row %)		Yes (n = 54) No. (Row %)		p-value	Adjusted Prevalence^b^	95% CI
Age, years	50.0^c^ (11.9)^d^		49.8^c^ (12.2)^d^		0.936	*	*
Male	37 (46.8)		42 (53.2)		0.431	53.5	46.9–60.0
Race					0.014		
AI/AN	1 (9.1)		10 (90.9)			65.6	41.2–89.9
Black	7 (63.6)		4 (36.4)			29.5	8.6–50.3
White	31 (44.3)		39 (55.7)			60.3	54.5–66.2
Other	4 (80.0)		1 (20.0)			18.1	0–48.2
Latino/Hispanic	5 (45.5)		6 (54.6)		1.00	–	–
Non-English primary language	4 (57.1)		3 (42.9)		0.696	–	–
Single	32 (42.7)		43 (57.3)		0.628	–	–
Homeless	9 (34.6)		17 (65.4)		0.260	–	–
Severity of Illness					0.001		
Extreme	24 (70.6)		10 (29.4)			57.7	44.1–71.3
Major	11 (29.7)		26 (70.3)			53.0	43.6–62.3
Moderate	8 (33.3)		16 (66.7)			54.5	44.3–64.6
Minor	0 (0)		2 (100)			^*^	^*^
Intubated	34 (91.9)		3 (8.1)		< 0.001	8.6	0–19.6
Admitting service					0.326	–	–
Medical ICU	18 (36.7)		31 (63.3)			–	–
Trauma ICU	13 (54.2)		11 (45.8)			–	–
Neurological ICU	12 (50.0)		12 (50.0)			–	–
Prior-year hospitalization with AWS	3 (15.8)		16 (84.2)		0.009	65.4	47.0–83.9
Positive serum/urine alcohol level^e^	20 (34.5)		38 (65.5)		0.022	52.7	45.4–60.1

*CI* confidence interval, *A/AN* American Indian/Alaska Native, * empty or unable to estimate,*–*  variable not included in multivariable regression analysis

^a^Assessment completed within 24 h of ICU admission

^b^Estimated using multivariable logistic regression including significant univariate associations (race, severity of illness, intubation, prior-year AWS, positive alcohol level) and other covariates selected a priori (age, sex) and the margins command in STATA; in the adjusted logistic regression analyses, only race and intubation were significantly associated with CIWA-Ar assessment (p = 0.018 and p < 0.001, respectively)

^c^Mean

^d^Standard deviation

^e^Assessment completed within 24 h of hospital presentation

Of the 54 patients with CIWA-Ar assessments, the majority (52%, n = 28/54) had scores indicating mild AWS (CIWA-Ar ≤ 8) requiring no additional pharmacotherapy. However, in the broader subset of 91 patients (94%) who had a RASS assessment within 24 h of ICU admission, a minority were considered optimally sedated (34%, n = 31/9, RASS -2 to 0), 37% were considered oversedated (n = 34/91, RASS -3 to -5), and 29% were considered undersedated or agitated (n = 26/91, RASS + 1 to + 4) [8].

## Discussion

In this sample of 97 ICU patients treated for AWS, nearly half (46%) were not monitored with the CIWA-Ar despite a hospital protocol specifying its use. CIWA-Ar monitoring varied across patient characteristics and was completed less often for patients who were intubated or identified as Black. This is the first study to evaluate clinical use of the CIWA-Ar in ICU patients.

Inconsistent application of the CIWA-Ar in ICU patients raises questions about its utility in the ICU. Although a high-visibility trial demonstrated symptom-triggered therapy using the CIWA-Ar reduced overall benzodiazepine exposure and treatment duration for patients with uncomplicated AWS [4], the CIWA-Ar requires patients to be oriented and communicative [2, 3]. Intubation (a mechanical obstruction to verbal communication) is a barrier to CIWA-Ar monitoring. Although use of the CIWA-Ar was appropriately deferred in all but 3 intubated patients in this study, AWS treatment monitoring is still warranted in the setting of intubation. The correct solution to this monitoring problem has not been identified.

This study has several limitations. It is the first to explore possible associations between patient characteristics and CIWA-Ar monitoring, thus a priori selection of potential confounders for the multivariable model was guided by limited literature. Significant results should be considered hypothesis-generating, especially given the small sample size. In particular, while the identified association between race and CIWA-Ar monitoring could reflect differences in care associated with racism, and/or biases and stigma associated with addiction [12–14], this association requires further exploration in larger samples of ICU patients. Additionally, a majority of patients included in the study (n = 92/97, 95%) were admitted primarily for management of acute medical/surgical illnesses rather than AWS. Signs/symptoms associated with these other conditions may have confounded the results of CIWA-Ar assessments. This is a limitation, in this study and in clinical practice, as previous research also suggests 96–98.5% of patients experiencing AWS in hospital settings are admitted for other acute indications [9–11]. Despite the inherent limitations of this single-hospital study design, it has important strengths: this is the first naturalistic evaluation of CIWA-Ar monitoring in ICU settings, data were derived from systematic chart reviews, and the study sample was defined such that timing of AWS treatment onset was standardized.

Results of this study support the feasibility of the American Society of Addiction Medicine’s recent suggestion that sedation measures such as the RASS be used, rather than the CIWA-Ar, to monitor AWS severity and response to treatment in ICU patients [2]. The RASS was completed in 94% of ICU patients with AWS in this study. Although never specifically evaluated/validated for management of AWS, the RASS has several strengths that make it potentially useful for AWS monitoring in ICUs: it is already widely used to support ICU care, it relies on provider assessment rather than patient interview, and it could identify both under- and over-treatment of AWS (i.e., bi-directionality). Future studies should assess the utility of the RASS for titrating AWS medications compared to other AWS severity scales that do not require patient self-report [15–23], emphasizing appropriate use in patients with wide-ranging characteristics to improve the quality of care for ICU patients with AWS.

## Data Availability

We do not have permission to release our data, which was collected via patient chart review and could compromise individual privacy.

## Abbreviations

AWS: Alcohol withdrawal syndrome
CIWA-Ar: Clinical Institute Withdrawal Assessment for Alcohol-Revised
ED: Emergency Department
ICD: International classification of diseases
ICU: Intensive care unit
RASS: Richmond Agitation-Sedation Scale
APR-DRG: All Patients Refined Diagnosis Related Groups
